# mTOR pathway in colorectal cancer: an update

**DOI:** 10.18632/oncotarget.1548

**Published:** 2013-12-05

**Authors:** Maria Giovanna Francipane, Eric Lagasse

**Affiliations:** ^1^ McGowan Institute for Regenerative Medicine, Department of Pathology, University of Pittsburgh School of Medicine, Pittsburgh, PA, USA.; ^2^ Ri.MED Foundation, Palermo, Italy.

**Keywords:** mTOR, colorectal cancer, cancer stem-like cells, personalized medicine

## Abstract

The mammalian target of rapamycin (mTOR) has emerged as a potential target for drug development, particularly due to the fact that it plays such a crucial role in cancer biology. In addition, next-generation mTOR inhibitors have become available, marking an exciting new phase in mTOR-based therapy. However, the verdict on their therapeutic efectiveness remains unclear. Here we review phosphatidylinositol-3-kinase (PI3K)/Akt/mTOR signaling as one of the primary mechanisms for sustaining tumor outgrowth and metastasis, recent advances in the development of mTOR inhibitors, and current studies addressing mTOR activation/inhibition in colorectal cancer (CRC). We will also discuss our recent comparative study of diferent mTOR inhibitors in a population of colon cancer stem cells (CSCs), and current major challenges for achieving individualized drug therapy using kinase inhibitors.

## INTRODUCTION

Classically, Akt has been viewed as the main upstream activator of mammalian target of rapamycin (mTOR). Indeed, activated Akt phosphorylates and inhibits tuberous sclerosis 2 (TSC2), allowing Ras homolog enriched in brain (Rheb) to accumulate in the GTP-bound state and trigger activation of the mTOR complex1 (mTORC1) pathway. mTORC1 is composed by mTOR, regulatory associated protein of mTOR (Raptor), mLST8/G-protein β-subunit like protein (GβL), RAS40 and Deptor. The activation of mTOR in mTORC1 leads to phosphorylation of ribosomal S6 protein kinase 1 (S6K1) and eIF4E-binding protein 1 (4E-BP1), mediators of protein translation and cell growth [1]. mTOR response to a wide range of intracellular (energy and stress) and extracellular (nutrients, growth factors, hormones) signals is mediated through these effectors. In response to nutrient and growth factor availability, mTORC1 suppresses autophagy, a process by which metabolically stressed cells recycle cytoplasmic components including organelles, to recover energy necessary for their survival. mTORC1 has also been recently identified as orchestrating anabolic cell growth by stimulating nucleotide synthesis through the pyrimidine synthesis pathway [2].

**Figure 1:**
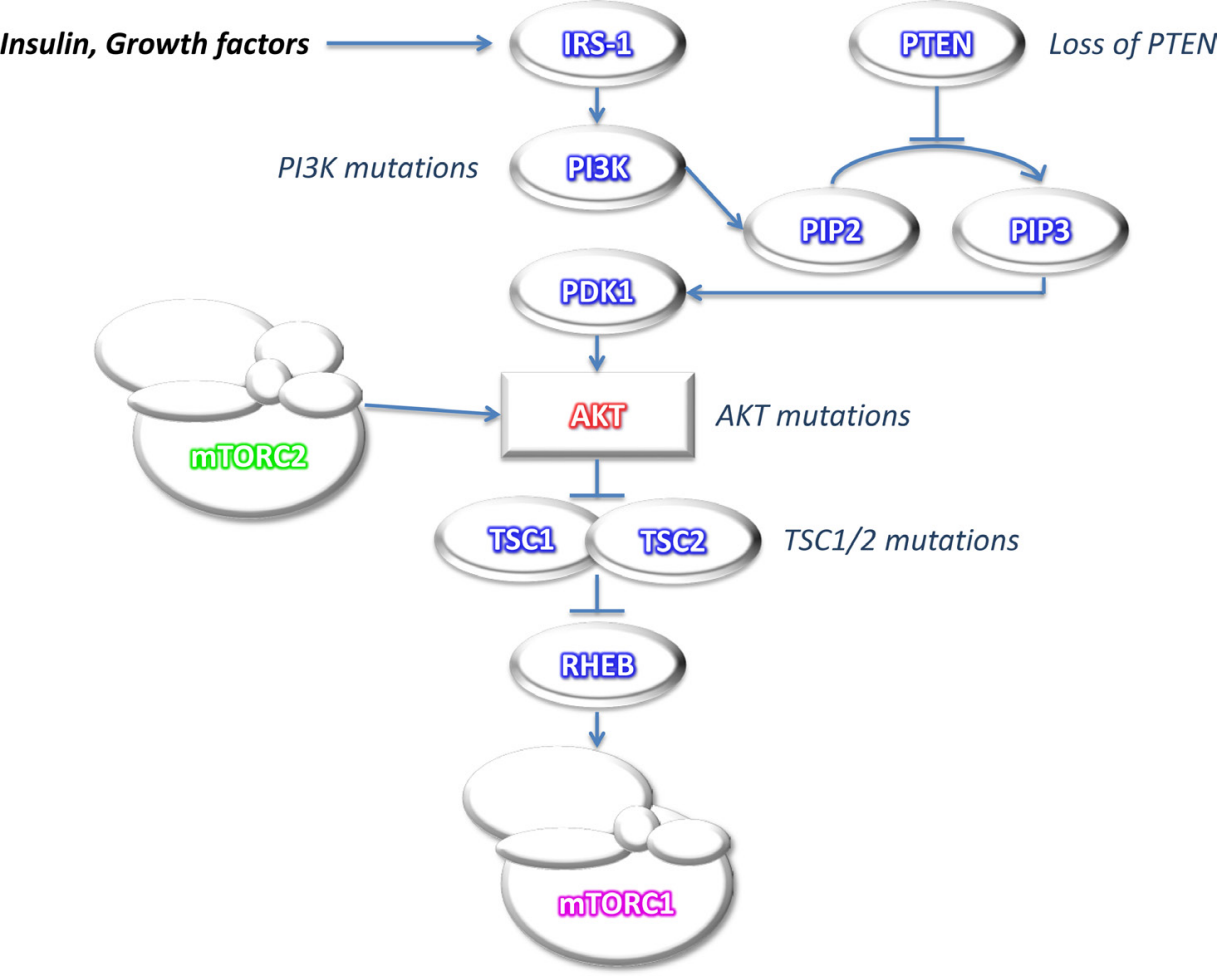
Simplified scheme of mTOR pathway activation Akt activation releases Rheb from the inhibitory effects of TSC1/2 thus allowing mTOR activation. Signaling defects upstream of mTOR in the PI3K/Akt/mTOR pathway (mutations in PTEN, PI3K, Akt and TSCs) lead to mTOR deregulation. Akt can also be a downstream effector of mTOR, due to mTOR association with different protein partners to form two functionally distinct signaling complexes, mTORC1 and mTORC2.

Different from mTORC1, mTORC2 is composed of mTOR, rapamycin-insensitive companion of mTOR (Rictor), mLST8/GβL, stress-activated-protein-kinase-interacting protein 1 (Sin1), proline-rich repeat protein-5 (PRR-5)/protein observed with Rictor-1 (Protor-1), and Deptor. The upstream regulation of mTORC2 is not well defined, although ribosome association appears to be a major, if not the sole, mechanism of mTORC2 activation [3]. mTORC2 plays an important role in cell survival, metabolism, proliferation and cytoskeleton organization, as it phosphorylates Protein Kinase Cα (PKCα), Serum/glucocorticoid-regulated kinase 1 (SGK1), as well as Akt, allowing for complete activation of Akt [4-7]. Akt is therefore both an upstream activator of mTORC1 and downstream effector of mTORC2 (Figure 1).

Because it plays such a crucial role in cancer biology, mTOR has emerged as a potential target for drug development. Several mTOR inhibitors have already gone through clinical trials for treating various cancers without great success. Nevertheless, the role of mTOR inhibitors in cancer therapy continues to evolve, as new compounds are synthetized.

In the present review we have focused on the role for mTOR in orchestrating key physiological and pathological processes, with a particular emphasis on colorectal cancer (CRC), which remains the second leading cause of cancer death in the United States [8].

### mTOR as a proto-oncogene

Although mTOR is frequently activated in human cancers, mutation of the mTOR gene has been found only occasionally [9-11]. This means that over-activation of the mTOR pathway is mostly due to signaling defects upstream of mTOR in the phosphatidylinositol-3-kinase (PI3K)/Akt/mTOR pathway. Mutations in PI3K alpha catalytic subunit kinase domain (PIK3CA) generally arise late in tumorigenesis, and can be identified in 32% of CRC tumors [12]. Loss of heterozygosity (LOH) and mutations in Phosphatase and tensin homolog (PTEN), a negative regulator of PI3K activity, have also been reported in CRC [13]. Both PIK3CA mutations and PTEN loss lead to mTOR over-activation. Although mutations in Akt genes are rarely found in CRC [14], a somatic missense mutation of Akt1 (E17K) in the pleckstrin homology (PH) domain resulting in constitutive association of Akt1 with the plasma membrane and Akt1 prolonged activation has been reported in CRC, which can lead to mTOR deregulation [15]. Similarly, although rare, germline TSC gene mutations, which have been associated with colonic hamartomatous polyps, account for 1% CRC, possibly through mTOR pathway triggering [16, 17] (Figure 1).

Several studies have attempted to characterize activating mutations in the mTOR gene [18-24]. These studies, mostly conducted using yeast, have demonstrated the impact of mutations on the function of mTOR and *in vitro* oncogenicity in some cases. However, the tumorigenic potential of the mTOR gene has only recently been established. By introducing mutations into evolutionarily conserved amino acid residues in major functional domains of human mTOR, Murugan *et al*. demonstrated that the mTOR gene is a proto-oncogene that possesses strong tumorigenicity when genetically activated [25]. Specifically, eight mutants in the HEAT repeats (M938T) and the FAT (W1456R and G1479N) and kinase (P2273S, V2284M, V2291I, T2294I, and E2288K) domains of mTOR were generated. These mutants showed increased protein kinase activities and activated the mTOR/p70S6K and Akt signaling pathways in human embryonic kidney cells (HEK293T). The kinase domain mutants which exhibited the greatest gains in activity, P2273S and E2288K were subsequently selected to explore the oncogenic potential of the mTOR gene in the mouse embryonic fibroblast cell line NIH3T3. mTOR mutant-expressing cells showed morphologic transformation and anchorage-independent growth, possessed invasive ability and *in vivo* tumorigenicity. This discovery of oncogenic mTOR mutations may facilitate the design of drugs targeting mTOR, as well as help predict their efficacy. For example, in yeast, resistance to Rapalogs has been associated with mutations in FK506 binding protein 12 (FKBP12) or the FKBP-rapamycin-binding (FRB) domain of TOR [26].

### mTOR's role in proliferation, differentiation and senescence

While emerging evidence supports a central role of the mTOR pathway in cell growth and cancer progression, increased mTOR activity can also play a role mediating the depletion of the epithelial stem cell compartment. Indeed, the aberrant activation of the mTOR pathway can paradoxically cause cells to undergo differentiation or senescence, thereby exiting the proliferative cell pool [27]. This concept is well demonstrated by the fact that persistent activation of mTOR by wingless-related MMTV integration site 1 (Wnt1) leads to accelerated epithelial stem cell senescence and premature aging in mice [28, 29]. Accordingly, inhibition of mTOR prevents the loss of proliferative epithelial progenitor stem cells upon radiation and enhances their tissue repopulating capacity [30]. Similarly, mTOR inhibition by Rapamycin enriches CD133^+^ subpopulations in liver tumor cells [31]. This enrichment is most likely achieved through blocking differentiation of the CD133^+^ subpopulations, enhancing apoptosis in the CD133^−^ subsets, and triggering the conversion of CD133^−^ to CD133^+^ cells. Thus, the maintenance of CD133^+^ cells *in vivo* by Rapamycin leads to high continuous tumorigenic potential in the context of liver cancer. These data suggest that mTOR signaling is involved in regulating the balance of proliferation and differentiation of cancer stem cells (CSCs) and that transient inhibition of mTOR can promote tumor re-emergence in certain tumor types via enrichment of CSCs.

The molecular mechanism(s) underlying these paradoxical effects of mTOR are not fully understood. It has been suggested that strong oncogenic signals (RAS, PI3K) concomitantly induce cell cycle arrest and activation of growth-promoting (i.e., anabolic) pathways such mTOR. Cell cycle arrest by itself is not yet senescence [32]. Nevertheless, in the presence of growth-stimulation, cell cycle blockage eventually leads to senescence. This mechanism by which arrested cells are converted to senescent cells has been named gerogenic conversion or geroconversion [33]. To avoid geroconversion, cancer cells must lose expression of cell cycle inhibitors, such as p53. Thus, cross-talk between p53 and the mTOR signaling pathways can determine whether stressed cells undergo apoptosis, reversible quiescence or irreversible senescence [34]. Inhibitors of mTOR can suppress geroconversion, protecting adult stem cells from undergoing premature cell senescence while simultaneously preventing their oncogenic transformation [35]. Amongst mTOR inhibitors, Rapamycin has been defined as a “longevity enhancer and cancer preventative agent” in the context of p53 deficiency [36]. Indeed, continuous treatment with Rapamycin or a novel Rapamycin formulation (Rapatar) delayed carcinogenesis in tumor-prone p53^+^/^−^and p53^−^/^−^mice respectively, most likely by slowing down the process of aging [37, 38]. Similarly, chronic treatment of mice with an enterically released formulation of Rapamycin (eRapa) delayed the onset and/or progression of neuroendocrine tumors in Rb1^+^/^−^ mice [39]. Likewise, hypoxia can decelerate geroconversion and extend lifespan. Indeed, not only does hypoxia arrests cell cycle, but also inhibits the mTOR pathway, thus preventing irreversible cellular senescence [40]. It turns out that in stem cell niches, stem cells might be protected from senescence and maintained in a quiescent status instead, thanks to the low oxygen levels which characterize stem cell niches [41]. Overall, these studies point out molecular differences in normal and cancer cells that can be exploited to prevent tumor growth without disrupting the function of normal tissues and cells.

**Figure 2:**
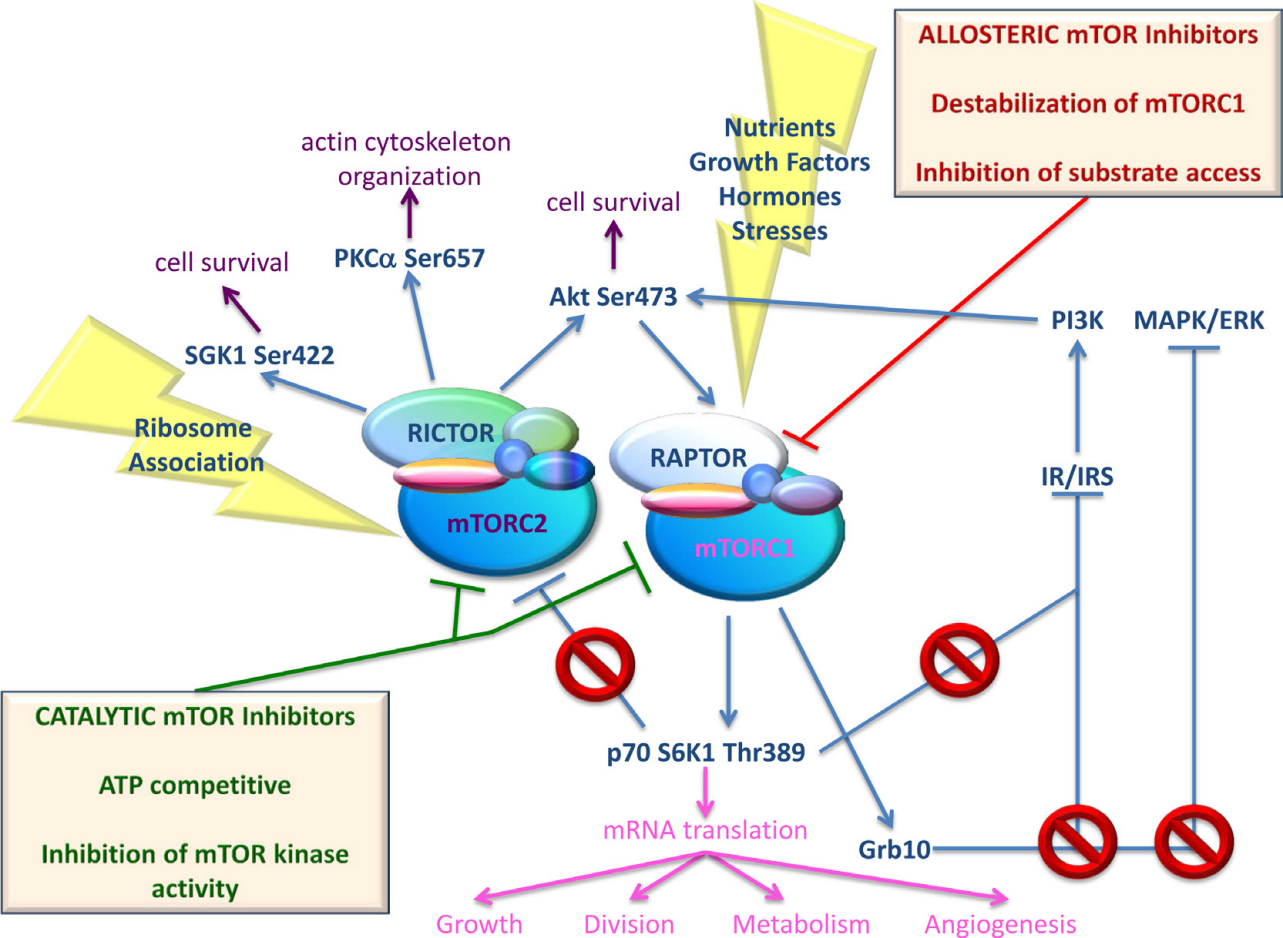
Simplified scheme of activators and effectors of both mTOR complexes, together with effects of mTOR inhibition using different mTOR inhibitors mTOR can be shared by two different complexes, mTORC1 and mTORC2. Phosphorylation of mTOR at Ser2448 and Ser2481 are indicative of mTORC1 and mTORC2 activation, respectively. mTORC1 can be activated by nutrients (amino acids, glucose), growth factors (Insulin, Insulin-like Growth Factor-1), hormones (leptin), and stresses (starvation, hypoxia, and DNA damage). Through mTORC1, these signals accelerate the synthesis of key proteins, involved in growth, division, metabolism and angiogenesis. mTORC1-activated p70 S6K1 and Grb10 mediate IRS-1 degradation, thus inhibiting PI3K/Akt activation. Grb10 also leads to negative feedback inhibition of MAPK/ERK pathway. Finally, activated p70 S6K1 inhibits mTORC2 signaling by phosphorylating Rictor on Thr1135. Allosteric mTOR inhibitors exert an incomplete inhibition of mTORC1 and are inactive against mTORC2 under short-term conditions. Moreover, they disrupt the mTORC1-dependent negative feedback loop to IRS-1/PI3K, MAPK/ERK and mTORC2. As a consequence, treatment with mTOR allosteric inhibitors often results in increased mTORC2 activity. mTORC2 functions upstream of Akt providing the critical second phosphorylation of Akt at Ser473, which is necessary for Akt full activation. mTORC2 also regulates cytoskeletal dynamics by activating PKCα, and regulates growth via SGK1 phosphorylation. The upstream regulation of mTORC2 is not well defined although ribosome association appears to be a major, if not the sole, mechanism of mTORC2 activation. Catalytic mTOR inhibitors are able to suppress activity of both mTORC1 and mTORC2 complexes, avoiding oncogenic signaling pathway activation

### Development of mTOR inhibitors: progress and challenges

Rapamycin, a macrolide antibiotic produced by *Streptomyces hygroscopicus*, was the first mTOR inhibitor discovered. More precisely, by exploiting Rapamycin's antifungal properties researchers were able to identify mTOR [42]. Guba *et al*. described Rapamycin antitumor effects for the first time in 2002, in their Nature Medicine paper [43]. Traditionally used as an immunosuppressant for organ transplants, Rapamycin was found to suppress tumor growth by inhibiting angiogenesis. Since then, additional inhibitors of mTOR function were synthesized with similar characteristics to Rapamycin, constituting the family of Rapalogs. Rapalogs, which also include Everolimus, Temsirolimus, and Ridaforolimus, bind FKBP12 and interfere with the FRB domain of mTOR. These compounds have gone through clinical trials as single agents for treating various cancers without great success. However, Temsirolimus and Everolimus were both effective against advanced renal cell carcinoma (RCC) [44]. While resistance to Rapalogs has been associated with mutations in FKBP12 or the FKB domain of TOR in yeast [26], in human, it has been mostly associated with KRAS and BRAF mutations [45]. Additional mechanisms of resistance include up-regulation of the Proviral integration site for Moloney murine leukemia virus (PIM) family of oncogenic serine/threonine kinases [46], oxidative stress [47], and over-expression of anti-apoptotic proteins [48]. Nevertheless, suppression of several negative feedback loops following mTORC1 targeting is mostly responsible for failure of mTOR inhibitors. Biopsies taken from patients affected by malignancies other than RCC, like colon and breast tumors, showed activated Akt following treatment with Rapalogs [49, 50]. Pro-survival rather than anticancer effects of Rapalogs likely results from disruption of the mTORC1-dependent negative feedback loop to mTORC2 and IRS-1/PI3K. Particularly, mTORC1-activated S6K1 phosphorylates Rictor and/or Insulin receptor substrate (IRS)-1, thus inhibiting mTORC2 and PI3K/Akt signaling, respectively [51, 52]. More recent findings indicate that mTORC1 also phosphorylates Growth Factor Receptor Bound Protein 10 (Grb10), leading to accumulation of Grb10 and negative feedback inhibition of PI3K and the Microtubule-associated protein kinase/Extracellular-signal regulated kinase (MAPK/ERK) pathway [53]. Thus, over-activation of upstream pathways following suppression of the above-mentioned feedback loops potentially counterbalances the antiproliferative effects of mTOR inhibitors. To overcome the detrimental consequences of feedback loop activation, a second generation of inhibitors, which compete with ATP in the catalytic site of mTOR, and inhibit both complexes, was developed [54]. Figure 2 depicts activators and effectors of both mTOR complexes, together with the effects of mTOR inhibition using different mTOR inhibitors. Table 1 and Table 2 provide a list of different ATP-competitive mTOR-selective or PI3K/mTOR dual inhibitors that have been validated in preclinical cancer studies. These studies showed inhibition of tumor growth in a number of xenograft models [55-147]. As shown in Table 1, ATP-competitive mTOR inhibitors have often been used in combination with ionizing radiation (IR) as well as many chemicals. Clinical trials have been completed for AZD8055, INK-128, OSI-027, NVP-BEZ23, and XL765. Additional clinical trials utilizing mTOR-inhibiting agents are ongoing.

**Table 1:** *In vivo* pre-clinical studies using ATP-competitive inhibitors of mTOR

Name	Xenograft models	Combinatorial Therapy	References
AZD2014	Patient-derived ER+ breast cancer	/	[55]
AZD8055					U87MG, BT474c, A549, Calu-3, LoVo, SW620, PC3, MES-SA	/	[56]
PTEN(+/-)LKB1(+/hypo)	/	[85]
Patient-derived primary HCC	SAHA	[57]
MTT	/	[86]
RD	AZD6244	[94]
INK-128	MDA-MB361	Lapatinib	[62]
KU-0063794	786-O	/	[130]
OSI-027				COLO 205, GEO	/	[65]
GEO, H292, Ovcar-5	Sunitinib (H292, Ovcar-5)	[128]
Jeko	/	[146]
HNSCC	Cetuximab	[126]
OXA-01	GEO	/	[128]
PP242									p190BCR-ABL, SUP-B15	Imatinib (p190BCR-AB), Dasatinib (SUP-B15)	[135]
8226 MM	/	[134]
LS174T	UO126	[105]
MDA-MB-231	/	[71]
LS174T, SW480	/	[61]
HS Sultan cells stably transfected with exogenous VEGF	/	[133]
Patient-derived colon cancer	/	[132]
U251	IR	[131]
DLD-1	Erlotinib	[129]
Torin-1		U87MG	/	[72]
Patient-derived colon CSCs	/	[171]
WYE-125132 (WYE-132)	MDA361, U87MG, A549, H1975, A498, 786-O	Bevacizumab	[76]
WYE-354	PC3MM2, U87MG	/	[75]

A panel of second-generation mTOR inhibitors is listed. Information regarding xenograft models as well as combinatorial drugs used in preclinical cancer studies for each mTOR inhibitor is provided. For information about combinatorial drugs, please consult legend of Table 2.

**Table 2:** *In vivo* pre-clinical studies using PI3K and mTOR dual inhibitors

Name	Xenograft models	Combinatorial Therapy	References
PC3M, U87MG	Temozolomide (U87MG)	[89]
BN472	/	[95]
HER2+ BT474	/	[96]
Tet-op-PIK3CA H1047R–CCSP-rtTA, LSL Kras G12D	ARRY-142886 (LSL Kras G12D)	[83]
Patient-derived primary pancreatic cancer	/	[81]
B16BL6	/	[147]
ENU-treated Tsc2+-	/	[93]
MM.1S	/	[91]
U87	/	[88]
CCSP-rtTA/Tet-op-K-Ras (FVB/N), H460	IR (H460)	[87]
EGFR T790M-L858R	AZD6244	[84]
RD/18	/	[92]
TC-71, RD/18	Vincristine	[90]
BC-1 PEL	/	[80]
786-0, A498	/	[82]
FL-18	/	[98]
Patient-derived glioblastoma (SJ28P3)	SL327	[99]
A172, patient-derived glioblastoma (SJ28P3)	/	[100]
Ptch+/-Hic+/-	NVP-LDE225	[101]
GS2	Chloroquine	[102]
DU145	Taxotere	[103]
LSL-K-ras(G12D/+)Pten(loxP/loxP)	/	[104]
LS174T	UO126	[105]
BEZ235 (NVP-BEZ235)																																																A549	RAD001	[106]
N87, MKN28, MKN45	/	[107]
JHH-7	/	[108]
A549	Chloroquine	[109]
786-0	Sorafenib	[110]
CAL62, TT	RAF265	[111]
GEM	/	[112]
AsPC-1	EMAP and/or Gemcitabine	[113]
Met-1, MCNeuA in the MKR mouse	/	[114]
LS174T, SW480	/	[61]
HEC-59, AN3CA	/	[60]
Tyr-HRas(G12V) Ink4a/Arf-/-, T11 OST, C3-TAg GEMM, MMTV-Neu GEMM	AZD6244	[121]
A549	Ganetespib	[115]
8505C	Paclitaxel	[58]
H295R	/	[59]
U-87 MG, 786-O	RAD001	[118]
Patient-derived PDAC	Panobinostat	[123]
U937 cells expressing doxycycline-inducible shRNAs against both Bcl-2 and Bcl-xL	ABT-737	[120]
PC-9/HGF	/	[122]
PC3	IR	[119]
4T1, 67NR	Dovitinib	[116]
LoVo, HCT116	5-FU or irinotecan	[117]
CNE2, HONE1	Cisplatin	[124]
RD	AZD6244	[94]
C4-2AT6	Docetaxel	[125]
PI-103														U87:ΔEGFR	/	[141]
U87MG, HCT116, PC3, MDA-MB-435, MDA-MB-468, SKOV3, IGROV-1	/	[66]
U251 MG	IR	[139]
p190 L-CFCs (hCD4+)	Imatinib	[143]
EC-vGPCR	/	[138]
U87MG	Temozolomide	[142]
Patient-derived neuroblastoma	Doxorubicin	[137]
518A2	Rapamycin	[144]
Gli36-EvIII-FmC, mNSC-S-TRAIL	/	[136]
PC-9 remixed with HGF high producing MRC-5 cells	Geftinib	[140]
SH-EP	TRAIL	[145]
SK-N-BE(2)	/	[67]
HGC27	5-FU	[69]
Huh7	Sorafenib	[68]
XL765				Patient-derived glioblastoma	Temozolomide	[77]
BxPC-3	Chloroquine	[78]
STS26T	Chloroquine	[79]
GH3	Temozolomide	[127]

A panel of second-generation mTOR inhibitors is listed. Information regarding xenograft models as well as combinatorial drugs used in preclinical cancer studies for each mTOR inhibitor is provided. SAHA and Panobinostat are histone deacetylase (HDAC) inhibitors; AZD6244, UO126, ARRY-142886, and SL327 are MEK inhibitors; Lapatinib, Cetuximab, Erlotinib, and Gefitinib are EGFR inhibitors; Sunitinib and Sorafenib are multitargeted kinases inhibitors; Imatinib and Dasatinib are tyrosine kinases inhibitors; Bevacizumab is an anti-vascular endothelial growth factor (VEGF) monoclonal antibody; Taxotere is a microtubule inhibitor; RAF265 is a BRAF inhibitor; Ganetespib is a heat shock protein 90 (Hsp90) inhibitor; ABT-737 is a Bcl-2 inhibitor; Dovitinib is an inhibitor of fibroblast growth factor (FGF), VEGF, and platelet-derived growth factor (PDGF) receptors; Vincristine and Paclitaxel are mitosis inhibitors; Irinotecan is a topoisomerase-I inhibitor; Temozolomide is an alkylating agent; NVP-LDE225 is a Smoothened (Smo) antagonist; Chloroquine is a lysosomotropic agent; Endothelial-monocyte activating polypeptide (EMAP) is a proinflammatory cytokine and a mediator of programmed endothelial cell death; 5-Fluorouracil, Cisplatin, and Doxorubicin are chemotherapy drugs; Tumor necrosis factor-related apoptosis-inducing ligand (TRAIL) is a cytotoxic protein and a mediator of programmed cell death.

mTOR kinase inhibitors (mTorKIs) do not cause Akt feedback activation observed with Rapalogs. However, Rodrik-Outmezguine *et al*. showed a biphasic effect of these drugs on Akt [148]. Inhibition of mTORC2 led to Akt Ser473 de-phosphorylation and a rapid but transient inhibition of Akt Thr308 phosphorylation and Akt signaling. Nevertheless, inhibition of mTOR kinase also relieves feedback inhibition of Receptor tyrosine kinases (RTKs) leading to subsequent PI3K activation and re-phosphorylation of Akt Thr308 sufficient to reactivate Akt activity and signaling. Importantly, similar mechanisms might also exist in tumor-associated stromal cells such as endothelial cells. Phosphorylation of Akt Thr308, Akt substrates [PRAS40, FoxO1 (forkhead box protein O1), and GSK-3 (glycogen synthase kinase–3)], and SGK substrate [NDRG1 (N-myc downstream regulated 1)] rebounds as early as 4 hours after adding mTorKIs in human umbilical vein endothelial cells (HUVECs), implying that PI3K is activated in response to mTOR inhibition in these cells [149]. Particularly, mTorKIs induce the expression of multiple RTK-encoding genes and signaling by promoting the de-phosphorylation of FoxO1, which eventually result in increased activation of PI3K. Thus mTorKIs may render endothelial cells more prone to PI3K inhibition, suggesting that combinations of PI3K and mTOR inhibitors might have synergistic effects and fully inhibit endothelial cell growth [149].

An additional limitation to the use of catalytic mTOR inhibitors is that they inhibit mTOR within the mTOR complexes and do not block intrinsic activity of mTOR-binding partners, which, although deprived of mTOR kinase activity, could continue conveying growth and survival signals in response to death stimuli. As opposed to Rapalogs and mTorKIs, P529 is a first-in-class allosteric inhibitor of mTORC1/mTORC2 that can dissociate both mTORC1 and mTORC2 complexes, thus overcoming possible activity of mTOR-binding partners. However, it is not clear whether P529 directly interacts with the mTOR complexes causing their dissociation. It may inhibit a chaperone, which assembles the mTOR complexes. Nevertheless, P529 was reported to inhibit tumor growth, angiogenesis, and vascular permeability [150], and to be effective in the treatment of prostate cancer [151, 152]. Moreover, P529 was explored as a therapeutic option for the treatment of keloid disease [153]. Aside from its association with cancer, deregulation of the mTOR pathway is in fact linked to several other diseases, including ocular, fibrotic, viral, skin and central nervous system diseases.

### mTOR signaling in colon: past and present evidence

mTORC1 is a major sensor of the organismal nutritional state. Indeed, caloric restriction lowers mTORC1 signaling in Paneth cells, a key constituent of the mammalian intestinal stem-cell (ISC) niche. Paneth cells, in turn, stimulate small-intestinal stem cells to proliferate [154]. Thus, mTOR inhibition can improve intestinal regeneration in patients affected by intestinal atrophy. While mTORC1 inhibition can increase the number and regenerative capacity of ISCs, excessive mTORC1 stimulation can lead to the onset of cancer. Indeed, the importance of mTORC1 pathway in intestinal polyp formation has been well described in several papers using the ApcΔ716 mice, a mouse model of familial adenomatous polyposis (FAP). Through these studies, mTORC1 was found to stimulate chromosomal instability (CIN) through anaphase bridge formation, enhancing, as a consequence, both tumor initiation and progression [155]. Similarly, *ex vivo* immunohistochemical studies on human colorectal adenomas and cancers confirmed that mTORC1 signaling occurs as an early event in the process of tumorigenesis, and participates in the progression of normal cells to a neoplastic phenotype [156], sustaining the bases of mTORC1-targeted drug development for therapy and prevention of colon polyps and cancers. Accordingly, Everolimus-mediated mTORC1 inhibition suppressed polyp formation and reduced mortality in ApcΔ716 mice [157]. However, blocking of a specific pathway may disrupt the balance between signaling pathways and enhance oncogenic signals. In that regard, in parallel with its cytostatic effect, mTORC1 inhibition by Rapamycin strongly increased MAPK kinase (MEK)/ERK activity, resulting in the appearance of a spindle morphology and higher invasiveness of KRAS-transformed intestinal epithelial cells (IECs) [158]. In this system, Rapamycin treatment also increased Bcl-2 levels, further indicating the need to develop new therapeutic drugs capable of overcoming the relief of feedback inhibition of pro-survival, pro-invasive and pro-metastatic pathways. Besides mTORC1, mTORC2 has been shown to be overexpressed in CRC and to play an important role in cancer biology. Down-regulation of mTORC2 reduced proliferation of colon cancer cell lines and inhibited the formation of tumor xenografts *in vivo* [159, 160]. Moreover both mTORC1 and mTORC2 complexes were shown to regulate epithelial-mesenchymal transition (EMT), motility, and metastasis of CRCs via RhoA and Rac1 GTPases, providing the rationale for including ATP-competitive mTOR-selective or PI3K/mTOR dual inhibitors for therapy of CRC patients [161]. Dual inhibition of PI3K and mTORC1/2 signaling by NVP-BEZ235 was shown to induce tumor regression in a genetically engineered mouse (GEM) model for sporadic CRC [112]. Consistent with this finding, a more recent study also demonstrated the efficacy of NVP-BEZ235 and of an additional catalytic mTOR inhibitor, pp242, in human colon cancer cell line xenografts [61]. Nevertheless, a remarkable intrinsic drug resistance of a large proportion of CRC cell lines to new-generation mTOR inhibitors, including both NVP-BEZ235 and pp242 compounds, was also described, warranting further studies [162]. By screening a panel of over 600 human cancer cell lines to identify markers of resistance and sensitivity to pp242, Ducker *et al*. found that KRAS mutations are responsible for conferring resistance to pp242 [132]. This resistance was specifically linked to changes in the level of phosphorylation of 4E-BP1, and was not evident in either a tumor with wild type KRAS or a tumor with a PIK3CA mutation in addition to KRAS. This and other studies [163] highlight the importance of monitoring the phosphorylation status of 4E-BP1 to assess responses to mTorKIs. Additional experimental evidence has revealed that pp242 treatment of a panel of CRC cell lines transiently inhibits Akt Ser473 phosphorylation while increasing the phosphorylation of epidermal growth factor receptor (EGFR) at Tyr1068 [129]. A parallel increase of Akt Ser473 and EGFR Tyr1068 in cells following pp242 treatment raises the possibility that, apart from KRAS mutations, EGFR phosphorylation, might also contribute to the incomplete inhibition of mTORC2 by pp242. Accordingly, the combination treatment of pp242 and erlotinib, an EGFR inhibitor, completely blocked both mTORC1 and mTORC2 activity, inhibited cell growth and suppressed the progression of CRC xenografts [129].

An additional and previously undescribed mechanism leading to Rapamycin resistance, based on 3-Phosphoinositide–dependent protein kinase-1 (PDK1)/Polo-like kinase 1 (PLK1)/Myc signaling, has also been reported in the context of CRC. Specifically, epigenetic loss of Protein phosphatase 2, regulatory subunit B, beta (PPP2R2B), occurring in >90% colorectal tumor samples, was indicated as a molecular event affecting the sensitivity of CRC to mTOR inhibitors [164]. On loss of PPP2R2B, Rapamycin triggers a compensatory Myc phosphorylation in PDK1-dependent, but PI3K and AKT-independent manner, resulting in resistance. Re-expression of PPP2R2B, genetic ablation of PDK1 or pharmacologic inhibition of PDK1 (using the small molecule BX912) abrogates Rapamycin-induced Myc phosphorylation, leading to Rapamycin sensitization. Additional studies revealed that PDK1 directly induces phosphorylation of PLK1, which in turn induces Myc phosphorylation and protein accumulation [165]. Importantly, the PLK1 inhibitor BI2536 worked in synergy with NVP-BEZ235 to induce robust apoptosis and tumor growth inhibition in CRC [165]. This study emphasizes the importance of epigenetic mechanisms in regulating oncogenic signaling and therapeutic response. Moreover, it indicates PPP2R2B may serve as a predictive marker for patient selection, whereas Myc phosphorylation can serve as a surrogate marker to evaluate the drug response.

### mTOR inhibitors for the ablation of colon cancer stem-like cells: future hopes

Over the last decade, proposed theories in cancer biology have drastically changed. Contrary to the longstanding clonal evolution model described by Nowell in the late 70's [166], the CSC hypothesis proposed that not every cell of the body could be the target of malignant transformation. The limited lifespan of a committed cell is likely shorter than the time necessary to accumulate tumor-inducing genetic changes. Therefore, cancer-initiating capability could be a unique feature of long-lived, self renewing stem cells [167]. Indeed, much experimental evidence indicates that stem cell transformation might be an early event in carcinogenesis. Transformation of normal stem cells through loss of APC is an extremely efficient route towards initiating intestinal adenomas [168]. The CSC hypothesis is neither a universal model for all cancers nor for all patients with the same disease. While some cancers have been hypothesized to initiate as a stem cell disease, they may then progress by clonal evolution of their CSCs, as CRC has been suggested to do through CIN [169].

To our knowledge, only one study investigated the effects of mTOR inhibitors in cancer stem-like cells so far. mTOR signaling was shown to be activated in colorectal cell line-derived spheres in serum-free medium [170]. Treatment with Rapamycin and pp242 diminished sphere-forming capacity as well as aldehyde dehydrogenase isoform 1 (ALDH1) activity. However, only pp242 suppressed the enrichment of ALDH^+^ cells induced by chemotherapy, thus highlighting an essential role of mTORC2 signaling in the maintenance of the CRC stemlike phenotype. Although this study further confirmed the importance of mTOR signaling in CRC, the authors did not perform sufficient functional experiments to assess the effect of mTOR inhibition on biological properties and tumorigenic potentials of CRC stem-like cells. Moreover, a controversy exists in the literature regarding the use of ALDH^+^ cells isolated from cancer cell lines as *in vitro* models for CSC study, further indicating the need to study the effect of mTOR inhibition using alternative methods to identify and characterize CSCs. Multiple cell-surface proteins have been proposed as potential candidate markers for colon CSCs, and our *in vitro* system, based on a feeder layer derived from rat mammary adenocarcinoma, efficiently enriches for these cells [169]. Recently, we analyzed colon CSCs for expression of major mTORC1/2 pathway components [171]. Colon CSCs exhibited unexpectedly low Akt signaling. Nevertheless, they showed mTORC2 activation. We indicated SGK1 as the possible main mTORC2 effector in colon CSCs. Akt hypo-phosphorylation and dependence of SGK family members for viability are known to occur most frequently in the context of wild-type PTEN, and helical PIK3CA mutations [172]. Future studies are needed to confirm whether this genetic signature can predict resistance or sensitivity of colon CSCs to different mTorKIs. Unfortunately, the prognostic and predictive value of common mutations in patients with CRC is controversial, due to a bias in research settings [173]. In our opinion, mTorKI resistance might also occur through less well-studied but equally important epigenetic mechanisms [174].

mTOR inhibitors affected colon CSCs differently, resulting in proliferation, induction of autophagy or apoptosis. The apoptosis-inducing mTOR inhibitor Torin-1 hindered growth, motility, invasion, and survival of CD326^+^/CD24^+^/CD49f^+^/CD29^+^ and CD326^+^/CD44^+^/CD166^+^ CRC subpopulations *in vitro*, and suppressed tumor growth *in vivo* with a concomitant reduction in vessel formation. Torin-1 also affected the expression of markers for cell proliferation, angio-/lympho-genesis, and stemness *in vivo*. Our study also indicated that although Torin-1 resistant clones can emerge, they are poorly tumorigenic, thus encouraging its potential use for CRC therapy. Because normal stem cells and CSCs share many traits, it seems reasonable to think that any therapy targeting CSCs may also destroy healthy tissues [175]. Through an innovative system based on the use of the mouse lymph node as an *in vivo* bioreactor [176], we showed that Torin-1 does not affect the survival of normal colon stem cells *in vivo*, suggesting its selectivity towards cancer cells. All these data support further development of Torin-1 for the clinical treatment of CRC.

### Concluding remarks: towards personalized medicine

mTOR is frequently activated in human cancers and is a commonly sought anticancer therapeutic target. Although direct substrates and downstream effectors of mTOR are well studied, the broad physiological role of mTOR implies that there are still downstream pathways to be identified. A more comprehensive understanding of the dynamics of mTOR signaling networks is therefore required for the design of safe and effective drug molecules targeting the mTOR pathway.

ATP competitive mTOR inhibitors have both advantages and disadvantages. Undoubtedly, they have a well-defined target, their biological effects are easy to be observed, and they transit quickly to clinical trials. On the other hand, they often have a high level of cross-reactivity with other kinases, are not pharmacologically tolerated, and easily lead to the development of drug resistance. Identifying biological factors that may predict efficacy or resistance to mTOR inhibitors is still challenging, since mTOR inhibitors may exert antitumor effects through multiple mechanisms of action. Moreover, crosstalk of the mTOR pathway with other pathways gives rise to compensatory loops for tumor cells to escape anti-tumor stimuli. This reveals the adaptive capabilities of oncogenic signaling networks and the limitations of monotherapy for inhibiting feedback-regulated pathways. Drug combinations targeting multiple pathways have been exploited to overcome this resistance but it is not clear which strategy will yield the greater therapeutic benefit. Additionally, the use of multiple drugs increases side effects and adverse reactions. In our study, a single mTOR inhibitor, Torin-1 was able to counteract CRC progression, supporting the rationale for its clinical testing [171]. Nevertheless, the precise mechanism by which Torin-1 acts remains to be elucidated. It must be noted that not all CRC patients could benefit from this therapy due to large individual variability in drug efficacy and safety. The observed discrepancy in outcome using different mTOR inhibitors in a population of colon CSCs is not only related to the difference of mTOR inhibitors in terms of pharmacokinetic and pharmacologic properties, but also to intrinsic variations in colon CSCs. A major goal of clinical pharmacology and pharmacogenomics is to establish phenotype-genotype associations that reveal drug response and toxicity. Unfortunately, although the impact of variations of the human genome sequence on the response to drug therapy has been increasingly considered in recent years, for complex diseases like CRC, it could be very difficult to determine unequivocally an exact phenotype or genotype that would predict therapy outcome. Genetic variations can result from single-nucleotide polymorphism (SNP), insertion, deletion, or duplication of DNA sequences. SNP of drug targets, drug-metabolizing enzymes, drug transporters, DNA repair enzymes, are likely to affect drug response [177]. In addition, non-genetic factors could contribute to variability in drug effects. Even if causal relations between variations and individual drug responses would be fully unraveled, the rapid development of resistance to targeted anticancer agents could represent a major challenge in targeted cancer therapy. Such resistance often results from secondary mutations of drug targets in cancer cells. Acquired resistance to imatinib in chronic myeloid leukemia (CML) and gastrointestinal stromal tumor (GISTs) through a secondary gene mutation in BCR-ABL and KIT genes, respectively, are examples of the adaptive capability of cancer cells to kinase inhibitors [178, 179]. Similarly, a dominant drug-resistant allele of mTOR (S2035T) eliminates the cellular actions of Rapamycin in the yeast *Saccharomyces cerevisae* [180]. It is not excluded that although primary mTOR mutations are barely observed in human cancers, the mTOR kinase could be hit by a secondary or even a *de novo* gene mutation in response to therapy as a self-defense mechanism.

In conclusion, although targeted therapy agents are increasingly available for clinical applications, many of these promising drugs have produced disappointing results when tested in clinical trials. Because most tumors are heterogeneous, a single drug regimen for patients with the same tumor type/histology is not always appropriate. Gene and protein signatures have been identified in several diseases, but this information struggles to be translated into clinically meaningful improvements. Nevertheless, these observations can help defeat the challenge of achieving individualized drug therapy, hopefully in the near future.
